# Preferred cataract surgery practices in Malaysia: a survey based study

**DOI:** 10.1186/s13104-023-06391-2

**Published:** 2023-06-22

**Authors:** Yong Zheng Wai, Yong Yuin Chong, Lik Thai Lim, Jan Bond Chan, Sudhashini Chandrasekaran

**Affiliations:** 1 Department of Ophthalmology, Kuala Lumpur Hospital, Kuala Lumpur, Malaysia; 2 Ampang Hospital, Ampang, Malaysia; 3 Faculty of Medicine, Universiti Malaysia Sarawak (UNIMAS), Kota Samarahan, Sarawak, Malaysia; 4 Universiti Malaysia Sarawak (UNIMAS), Kota Samarahan, Sarawak, Malaysia; 5 International Specialist Eye Center, Kuala Lumpur, Malaysia; 6 Department of Ophthalmology, Faculty of Medicine, University of Malaya, Malaya, Malaysia

**Keywords:** Cataract surgery, Phacoemulsification, Cataract surgery practice trend

## Abstract

**Background:**

To describe the preferred cataract surgery practices among Malaysian ophthalmologists and medical officers in comparison with the recommended practices.

**Methods:**

An online questionnaire was sent out in April 2021, to Malaysian Ophthalmologists and medical officers who perform cataract surgeries. The questions were focused on the preferred cataract surgery practices of the participants. All data obtained were collected, tabulated and analysed.

**Results:**

A total of 173 participants responded to the online questionnaire. 55% of the participants were within 31–40 years of age. 56.1% preferred peristaltic pump over venturi system. 91.3% of participants practised povidone iodine instillation to the conjunctival sac. With regards to the main wound incision, more than half of the surgeons (50.3%) preferred fixed superior incision and 72.3% of them preferred 2.75 mm microkeratome blade. Most of the participants (63%) were inclined towards C-Loop clear intraocular lens (IOL) with a single-handed push preloaded system. 78.6% of the surgeons routinely use carbachol in their cataract surgery.

**Conclusions:**

This survey provides some insight into the current practices among Malaysian ophthalmologists. Most of the practices are in line with international guidelines for preventing postoperative endophthalmitis. This article could help trainees and ophthalmologists benchmark and observe the common cataract surgery practices among their seniors and peers in Malaysia.

**Supplementary Information:**

The online version contains supplementary material available at 10.1186/s13104-023-06391-2.

## Background

Age-related cataracts remain the leading cause of blindness in middle and low-income countries [1]. Cataract extraction is the most commonly performed eye operation in the world [2]. Cataract surgery practice patterns have changed over the past years with better surgical outcomes and safety. Many countries like the USA, Canada, Japan, New Zealand, Korea, Jordan and Thailand have reported their current preferred cataract surgical practice [3–8].

Korean Society of Cataract and Refractive Surgery reported their latest cataract surgical practice in 2018 with an increasing trend of premium intraocular lens (IOL), optical biometry and topical anaesthesia usage [7]. On the other hand, New Zealand cataract and refractive surgery survey in 2007 revealed higher use of subtenon anesthesia, others results were similar with American Society of Cataract and Refractive Surgery members [6].

The last reported Malaysian cataract surgery practice pattern was dated back in 2014 [9]. From the previous study we noticed there was a paradigm shift from extracapsular cataract extraction (ECCE) to phacoemulsification surgery [9]. The current preferred practice among eye surgeons might have changed from 7 years ago. An understanding of the current trend is essential because of the rapidly evolving nature of cataract surgery. This survey may also help as a guide for the new cataract surgeon, on what is the common practice among their peers and seniors.

In this study, we surveyed the common cataract surgical practices among Malaysian ophthalmologists and ophthalmology medical officers in 2021.

## Methods

In April 2021, questionnaires with 19 multiple choice questions regarding cataract surgery practices, were sent to all ophthalmologists and ophthalmology medical officers who perform cataract surgeries through MSO email, ministry of health WhatsApp groups and university WhatsApp groups. Participants were expected to answer the questionnaire with their common practices in cataract surgery.

The information from these questionnaires was recorded and analysed. Data of interests included gender, age, current position, sector of service, phacoemulsification machine system, pre-operative povidone iodine instillation, type of local anaesthesia, location of the main incision, size of microkeratome, type of blade for paracentesis, usage of vision blue, techniques to loosen cataract, techniques for phacoemulsification, type of intraocular lens (IOL), type of preloaded lens system, the habit of polishing capsular bag, usage of Miostat (Carbachol) and type of intracameral antibiotics. IBM SPSS Statistics version 25.0 was used for statistical analysis.

## Results

A total of 173 participants responded to the online survey from the members of Malaysian Society of Ophthalmology. Demographic information is illustrated in Table 1.

**Table 1:** Demography of surgeons

	Frequency	Percentage (%)
Gender		
Male	73	42.2
Female	100	57.8
**Age Group**		
Less than 30	3	1.7
31–40	96	55.5
41–50	48	27.7
Above 50	26	15.0
**Qualification**		
Medical Officer	9	5.2
Master Student	21	12.1
Gazetting Specialist	15	8.7
Specialist/ Consultant	128	74.0
**Service sector**		
Private University	1	0.6
Public University	30	17.3
Armed Force	3	1.7
Private Eye Clinic	32	18.5
Private Hospital	31	17.9
Government Hospital	76	43.9
Place of practice (State)*		
Northern Region	26	15.0
Central Region	87	50.2
East Coast Region	8	4.6
Southern Region	31	17.9
East Malaysia (Borneo)	21	12.1

*Northern Region: Perlis, Kedah, Penang, Perak; Central Region: Kuala Lumpur, Selangor; East Coast Region: Pahang, Kelantan, Terengganu; Southern Region: Negeri Sembilan, Malacca, Johor; East Malaysia: Sarawak, Sabah.

### Pre-operative preparation and anaesthesia

Povidone iodine was instilled in the conjunctival sac before cataract surgery by 93.1% of the surgeons. Solely topical anaesthesia was used by 42.8% of respondents, followed by Topical anaesthesia plus intracameral (41%) and lastly subtenon anaesthesia (16.2%).

### Main wound and paracentesis construction

Fixed superior incision (50.3%) was the preferred location for main wound construction, followed by incision on the steep axis (29.5%) and the remaining 20.2% chose fixed temporal incision. 72.3% of the respondents created the main wound by using 2.75 mm microkeratome blade and only 6.4% used 2.2 mm microkeratome blade. The majority of surgeons (66.5%) made paracentesis by using a 15-degree blade, the remaining 33.5% chose microkeratome to create paracentesis.

### Cataract surgery techniques

**Fig. 1:**
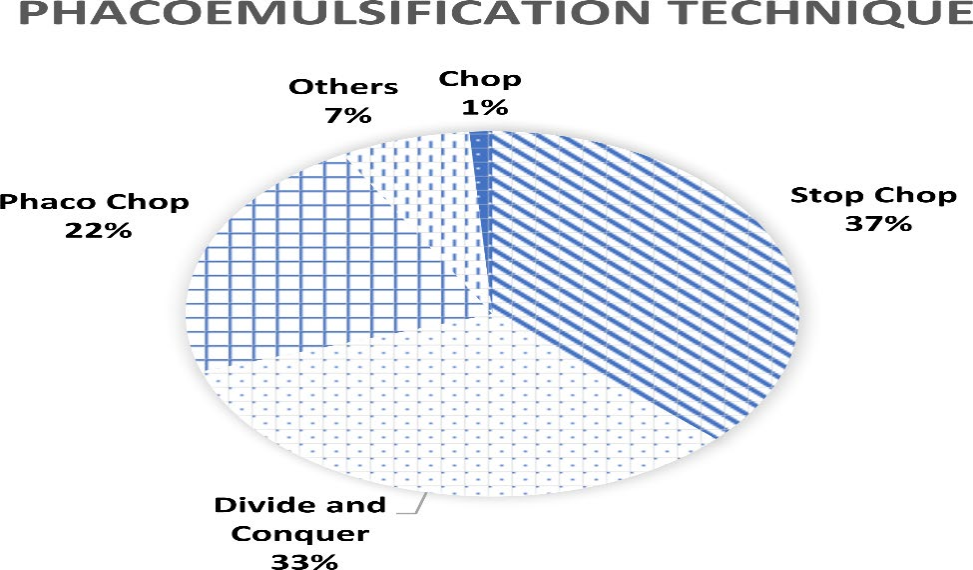
Phacoemulsification technique among Malaysian ophthalmologists and medical officers

In terms of phacoemulsification machine systems, 56.1% of surgeons preferred peristaltic pumps over venturi systems (43.9%). Most surgeons (68.8%) preferred to use hydrodissection to loosen the cataract and 31.2% used both hydrodissection and hydrodelineation to separate the cataract. Phacoemulsification technique is illustrated in Fig. 1. 89% of respondents used coaxial irrigation-aspiration during cortical matter aspiration and remaining 11% used bimanual irrigation-aspiration. Only 46.8% of surgeons routinely polish the capsular bag.

### IOL designs and implantation

Clear IOL (69.4%) was preferred to the yellow lens (30.6%). Majority inclined towards C-loop IOL design (68.8%) rather than plate haptic design (28.9%). Most respondents preferred a pre-loaded IOL (78%). Among pre-loaded IOL implantation, a single-handed push system (63%) was more popular compared to a double-handed screw (37%).

### Intraocular drug usage

**Fig. 2:**
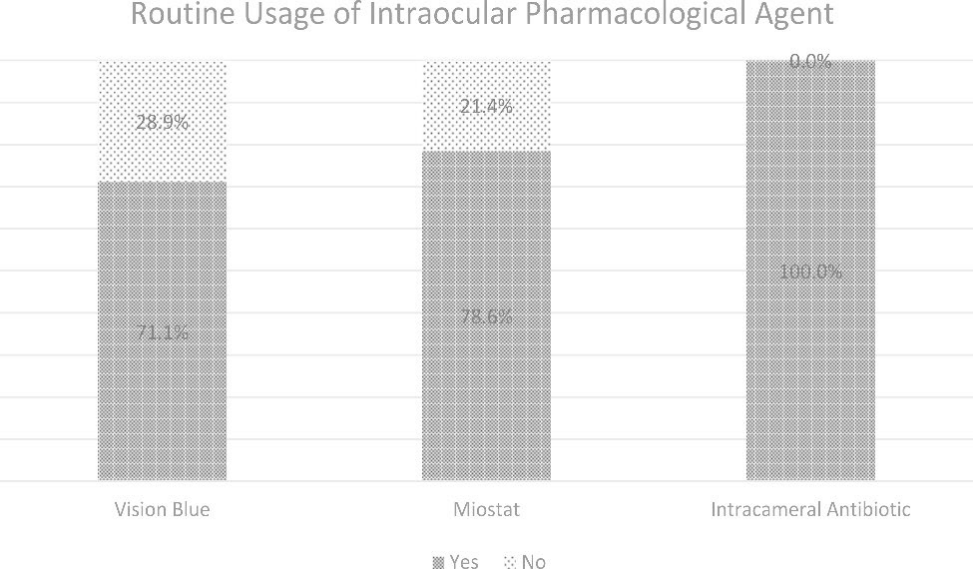
Usage of intraocular pharmacological agent

Intracameral antibiotics were routinely applied towards the end of the surgery. Cefuroxime remained the commonest intracameral antibiotic (60.7%), followed by Moxifloxacin (36.4%) and Levofloxacin (2.9%). Vision blue and Miostat (carbachol) usage are shown in Fig. 2.

## Discussions

This survey summarizes the current preferred cataract surgery practices among Malaysian ophthalmologists and medical officers. Most of the respondents (74%) were specialists or consultant ophthalmologists.

The pros and cons of pre-operative povidone iodine instillation to prevent endophthalmitis has been published a few years ago [10]. Malaysia post-operative endophthalmitis (POE) was reported as low as 0.08%, and this could be a result of routine instillation of povidone-iodine before cataract surgery [11]. The European Society of Cataract and Refractive Surgeons (ESCRS) recommends surgeons to instill 5–10% of povidone iodine to the cornea and conjunctival sac for at least 3 min to reduce postoperative endophthalmitis rate [10]. However, it is not without risk as Ridder et al. reported that povidone-iodine 5% significantly decrease corneal epithelial integrity and increase subjective complaints of ocular discomfort from the patients [12]. Thus, the risk and benefit of povidone-iodine instillation have to be taken into consideration (should add here what is the recommended practice for povidone iodine).

Topical anaesthesia plus intracameral anaesthesia remained the most popular local anaesthesia, with a marked increase from 58.8% (2011) to 83.8% (2021) (how did you get the 2011 data?) [9]. This figure was similar to a Korean’s survey [7]. Subtenon anaesthesia reduced from 36.7% (2011) to 16.2% (2021). This reduction could be attributed to the reducing number of extracapsular cataract extraction (ECCE) surgeries.

New Zealand and Korean ophthalmologists have predilection over the temporal corneal incision, ranging between 57 and 71.2% [6, 7]. In contrast, most of the respondents of our survey preferred fixed superior corneal incision (50.3%). Only 20.2% chose fixed temporal corneal incision. A majority (72.3%) used 2.75 mm microkeratome to create the main corneal incision. New Zealand’s survey had a similar result, where 57% of them used 2.75 mm to create the main wound [6]. 33.5% of respondents used microkeratome to create the paracentesis wound. This is likely to cut overall cost of the cataract surgery.

Phacoemulsification machines can be broadly divided into 2 different systems: peristaltic pump and venturi system. Each has its pros and cons with a similar safety profile in terms of risk for posterior capsular rent [13]. In this survey, the peristaltic pump was more popular (56.1%) compared to the venturi system (43.9%). There is a different approach in nucleofractis technique. The 2 main techniques were stop-chop (37%) and divide and conquer (32.9%). Phaco-chop technique was the third most common technique (23.2%).

Coaxial aspiration-irrigation was commonly used in many centres as almost all centres have such aspiration-irrigation tip. This might be the cause for its popularity among Malaysian ophthalmologists. Bimanual aspiration-irrigation has better accessibility to all areas of the capsular bag compared to the coaxial tip [14]. Otherwise, there is no significant difference in terms of posterior capsular formation among these 2 aspiration-irrigation techniques [14].

The benefits of yellow IOLs versus clear IOLs have been extensively studied. The yellow lens can affect the perception of luminance and possibly disrupt circadian rhythm compared to clear IOL [15, 16]. Most of the participants preferred clear IOL over yellow IOL. Plate haptic has better stability and has less risk for decentration and tilt compared to C-loop haptic and both of the haptic designs have a similar risk for posterior capsular opacification [17–19]. Despite plate haptic having this added advantages, C-loop haptic remained more favourable among Malaysian ophthalmologists.

Preloaded IOL delivery system has proven to shorten the surgery time and increase economic efficiency [20]. Merits of preloaded system includes prevention of IOL setting errors, potential IOL damage and elimination of variability in manual loading [21]. Double handed screw delivery system require surgeon to use both hands, single-handed push allow surgeon to use the second hand to stabilize the eye. Hence more participants preferred single-handed push preloaded system.

Prophylactic intracameral antibiotic has been proven to reduced incidence of endophthalmitis. ESCRS recommended intracameral cefuroxime injection at the end of cataract surgery [10]. Other antibiotics such as moxifloxacin and vancomycin showed similar efficacy [22]. All 3 intracameral antibiotics (cefuroxime, moxifloxacin and levofloxacin) have no safety issues when used intraocularly [23]. Probably due to the long history of safety profile of intracameral cefuroxime, it is the most popular antibiotic of choice.

Surprisingly more than a quarter of participants inject intracameral carbachol (Miostat) in their cataract surgery. There is no consensus to encourage surgeons to inject carbachol routinely during the surgery. Even though the side effect of carbachol is rare, it should be used only in selected cases. Routine usage of carbachol will increase the cost of the surgery and lengthen the surgical time, as well as reducing the theoretical risk of toxic anterior segment syndrome (TASS).

### Limitations

This survey did not cover every ophthalmologist in Malaysia. Some of the participants refuse to respond to the online survey. Besides that, cataract surgery is a dynamic event in which surgeons might change their preferences on a case-to-case basis. Due to this, some participants might have more than 1 answer to some questions. However, they can only choose 1 single answer for all the questions which might not reflecting the real-world condition.

## Conclusion

This study provides some insight into the current practices among Malaysian ophthalmologists. This article could help trainees and ophthalmologists to benchmark and observe the common cataract surgery practices in Malaysia.

## Electronic supplementary material

Below is the link to the electronic supplementary material.

Supplementary Material 1

## Data Availability

All data and materials used to support the finding of this study are available from the corresponding author upon request.

## Declarations

This data has not previously been published in elsewhere or under consideration of publication in another journal. This questionnaire was developed for this study. This questionnaire has been uploaded as supplementary file.

## List of abbreviations

IOL: Intraocular lens
ESCRS: European Society of Cataract and Refractive Surgeons
POE: Post-operative endophthalmitis
ECCE: Extracapsular cataract extraction
TASS: Toxic anterior segment syndrome
