# Supplementary material for: Preferred cataract surgery practices in Malaysia: a survey based study
Source: BMC Res Notes. 2023 Jun 22;16:116. doi: 10.1186/s13104-023-06391-2 (PMC10288741; doi:10.1186/s13104-023-06391-2)
Supplement: Supplementary file 1 — Supplementary Material 1 [file 13104_2023_6391_MOESM1_ESM.docx]

Questionnaire Data Collection Form:

1. Gender:

-Male

-Female

1. Age group:

<30 years old

31-40 years old

41-50 years old

>50 yearas old

1. Current position:

-Medical Officer

-Master Students

-Gazetting Specialist

-Specialist/Consultant

1. Which sector are you in practising in?

-Government Hospital

-Private Hospital

-Private eye clinic

-Public University

-Private University

-Armed Forces

1. Which state are you in?

-Perlis

-Kedah

-Kelantan

-Penang

-Perak

-Pahang

-Terengganu

-Selangor

-Kuala Lumpur

-Negeri Sembilan

-Melaka

-Johor

-Sarawak

-Sabah

1. Which phacoemulsification machine system do you prefer?

-Peristaltic pump

-Venturi system

1. Do you apply povidone iodine in the conjunctival sac?

-Yes

-No

1. Which is your preferred / commonly used local anaesthesia?

-Topical anesthesia

-Intracameral anesthesia

-Subtenon anesthesia

1. Where do you place your main incision?

-Fixed superior incision

-Fixed temporal incision

-Based on the steep axis

1. Which microkeratome blade do you commonly use?

-2.75mm

-2.4mm

-2.2mm

-Others

1. Which blade do you use to make your paracentesis?

-15-degree blade

-Microkeratome blade

1. Do you commonly use vision blue?

-Yes

-No

1. Which technique do you prefer loosen and separate the cataract to facilitate its removal?

-Hydrodissection

-Hydrodelineation

-Both

1. Which technique do you use for phacoemulsification?

-Divide and conquer

-Stop chop

-Phaco chop

-Divide and conquer + phaco chop

-Chop

1. Which type of lens do you routinely implant?

-Clear

-Yellow lens

1. Which type of lens design do you prefer?

-C-loop IOL

-Plate haptic

1. Which type of lens do you prefer?

-Pre-loaded IOL

-Unloaded IOL

1. Which type pre-loaded lens system do you prefer?

-Single-handed push

-Double-handed screw

1. How do you perform cortical matter aspiration?

-Bimanual irrigation-aspiration

-Coaxial irrigation-aspiration

1. Do you routinely polish the capsular bag?

-Yes

-No

1. Do you routinely use Miostat (carbachol)?

-Yes

-No

1. Which type of intracameral antibiotics do you use?

-Cefuroxime

-Moxifloxacin

-Levofloxacin
